# Supplementary material for: Optimization of inner panel thickness for enhanced stiffness and vibration control in car door assemblies
Source: PLoS One. 2025 Nov 13;20(11):e0331318. doi: 10.1371/journal.pone.0331318 (PMC12614550; doi:10.1371/journal.pone.0331318)
Supplement: S1 Data — (DOCX) [file pone.0331318.s001.docx]

**Table 3: Experimental Test and FE Analysis Frequency Comparison**

**(Door Assembly (26.7kg): Window Glass Opening Effect)**

| **Modal Test - Mode Shape Description** | **Modal  Test** | **T10** | **T12** | **T14** | **T16** | **T18** | **T20** | **T22** | **T24** | **Optimized T15=1.52** |
| --- | --- | --- | --- | --- | --- | --- | --- | --- | --- | --- |
| **Mass (kg)** | **26.6** | **24.12** | **25.53** | **26.70** | **28.34** | **29.74** | **31.15** | **32.56** | **33.96** | **27.78** |
| 1^st^ Lateral Bending | 26.2 | 25.9 | 26.0 | 26.1 | 26.2 | 26.3 | 26.4 | 26.4 | 26.5 | 26.2 |
| 1^st^ Vertical Bending | 32.4 | 35.3 | 35.7 | 36.0 | 36.2 | 36.4 | 36.5 | 36.6 | 36.7 | 36.1 |
| 2^nd^ Vertical Bending | 38.6 | 39.0 | 40.1 | 40.8 | 41.4 | 41.8 | 42.1 | 42.3 | 42.4 | 41.3 |
| 2^nd^ Lateral Bending | 43.5 | 39.8 | 41.6 | 42.5 | 43.5 | 44.3 | 44.9 | 45.5 | 46.1 | 43.2 |
| 1^st^ Torsional Bending | 49.4 | 49.7 | 50.5 | 51.0 | 51.6 | 52.0 | 52.6 | 53.4 | 54.4 | 51.4 |
| 2^nd^ Torsional Bending | 55.4 | 52.6 | 54.5 | 56.0 | 58.4 | 58.5 | 60.4 | 61.3 | 61.5 | 57.1 |

**Table 4: Minimum Dynamic Stiffness at Critical Locations Between 20 Hz - 60 Hz**

| Minimum Dynamic Stiffness (N/m) at Critical Locations between 20 Hz - 60 Hz | | | | | | | | | | | | | | | | |
| --- | --- | --- | --- | --- | --- | --- | --- | --- | --- | --- | --- | --- | --- | --- | --- | --- |
| Model | D1 | D2 | D3 | D4 | D5 | D6 | D7 | D8 | D9 | D10 | D11 | D12 | D13 | D14 | D15 |  |
| T10-1.0mm | 70.70 | 75.32 | 80.27 | 77.60 | 200.81 | 42.87 | 35.29 | 176.22 | 108.73 | 63.72 | 54.69 | 84.84 | 5.65 | 15.39 | 16.92 |  |
| T12-1.2mm | 73.53 | 85.34 | 91.59 | 108.81 | 219.25 | 44.34 | 36.38 | 137.72 | 137.41 | 66.04 | 56.20 | 86.62 | 6.91 | 17.81 | 19.26 |  |
| T14-1.367mm | 69.83 | 91.22 | 101.44 | 110.88 | 233.77 | 45.43 | 37.49 | 148.40 | 161.38 | 78.90 | 67.31 | 95.72 | 7.53 | 18.31 | 24.63 |  |
| T16-1.6mm | 72.58 | 95.57 | 138.26 | 139.09 | 263.69 | 46.71 | 38.87 | 167.52 | 169.91 | 85.76 | 76.56 | 119.21 | 11.19 | 16.71 | 22.60 |  |
| T18-1.8mm | 68.29 | 87.14 | 131.35 | 144.91 | 267.09 | 47.59 | 39.91 | 189.06 | 177.75 | 83.54 | 81.65 | 161.05 | 12.76 | 15.08 | 19.78 |  |
| T20-2.0mm | 73.02 | 92.39 | 143.90 | 151.27 | 269.66 | 48.23 | 40.89 | 233.90 | 185.93 | 95.58 | 94.22 | 168.20 | 14.78 | 14.51 | 19.02 |  |
| T22-2.2mm | 87.42 | 104.70 | 150.75 | 158.02 | 275.67 | 48.90 | 41.88 | 248.12 | 194.40 | 88.87 | 95.18 | 175.55 | 24.97 | 17.85 | 22.82 |  |
| T24-2.4mm | 83.65 | 101.70 | 151.82 | 165.08 | 283.44 | 49.55 | 42.87 | 261.97 | 203.10 | 96.08 | 104.53 | 183.08 | 39.92 | 15.57 | 19.95 |  |
| T15-1.52mm | 62.09 | 81.91 | 117.75 | 136.95 | 246.82 | 46.30 | 38.42 | 153.75 | 166.90 | 81.71 | 70.55 | 121.30 | 9.26 | 15.04 | 20.08 |  |
