# Supplementary material for: Optimization of inner panel thickness for enhanced stiffness and vibration control in car door assemblies
Source: PLoS One. 2025 Nov 13;20(11):e0331318. doi: 10.1371/journal.pone.0331318 (PMC12614550; doi:10.1371/journal.pone.0331318)
Supplement: Attachment — Submitted filename: PONE_answer to reviewers.docx [file pone.0331318.s003.docx]

**Responses to the Review Comments**

| ***Reviewer #1*** | | | |
| --- | --- | --- | --- |
| **Sr. No.** | **Instructions and queries** | **Corrections/modifications made as per instructions and queries** | |
|  |  | **Status** | **Detailed description (All changes are shown by blue colour)** |
| 1 | “…the system-damping effect is ignored to avoid numerical complexity,” A big simplification. Please justify this assumption. | Required changes are done.  [Page no. 7; Line no. 159] | We sincerely thank the reviewer for pointing this out. Yes, as correctly observed, Equation (1) has been simplified to Equation (2) by neglecting the system damping component. The justification for this assumption is as follows:   - This approach is a standard and widely accepted practice in linear modal analysis, particularly when the primary objective is to compute the natural (modal) frequencies of the system. - For most engineering systems, damping is relatively small (i.e., underdamped systems), and its influence on the calculated natural frequencies is minimal. The damped natural frequency ω_d_  is related to the undamped natural frequency ω_n_ by the expression:      - Including damping in the initial formulation would increase numerical complexity without significantly improving the accuracy of the frequency estimation for the intended analysis. |
| 2 | Accelerometer Characteristics (Sensitivity, Mass and Dynamic Range). Please describe. | Required changes are done.  [Page no. 13 & 14; Line no. 306 to 316] | We appreciate the reviewer’s observation. The accelerometer used in the study is a PCB make model 356A01. Its detailed specifications are as follows:   - **Sensitivity (±20%)**: 0.51 mV/(m/s²) - **Mass**: 1.0 g (sensor only), approximately 4.2 g with cable - **Measurement Range (Dynamic Range): ±9810 m/s² peak** - **Frequency Range (±5%): 2 to 5000 Hz** - **Resonant Frequency: ≥ 50 kHz**   These details have now been incorporated into the revised manuscript for clarity. |
| 3 | As the accelerometer will typically have an increase in sensitivity at the high frequency end due to its resonance, its output will not give a true representation of the vibration at the measuring point at these frequencies. Please describe. | Required changes are done.  [Page no. 13 & 14; Line no. 306 to 316] | Thank you for your observation. The accelerometer used in our study has a resonance frequency above 50 kHz, which is significantly higher than our frequency range of interest (0–100 Hz). Since we are operating well below the resonance region, the accelerometer maintains a flat frequency response within our measurement range, and no amplification due to resonance effects is observed. Therefore, the concern regarding increased sensitivity at high frequencies does not apply in our case, and the accelerometer output accurately represents the vibration at the measuring point within the specified frequency range. |
| 4 | The method of mounting the accelerometer to the measuring point is one of the most critical factors in obtaining accurate results from practical vibration measurements. Mounting the Accelerometer. Please describe. | Required changes are done.  [Page no. 13; Line no. 317 to 318] | We appreciate the reviewer’s observation regarding the importance of accelerometer mounting in achieving accurate vibration measurements. In our experimental setup, the accelerometers were mounted using a two-step method: initially, a strong adhesive was applied to ensure firm contact and minimize any relative motion between the sensor and the surface. Subsequently, one-sided self-adhesive paper tape was used to mask and secure the accelerometer in place, providing additional stability and protection against environmental disturbances. This mounting approach was chosen to ensure reliable signal transmission and repeatability of measurements. |
| 5 | There are two basic types of filter used for the frequency analysis vibration signals. The constant bandwidth type filter, where the filter is a constant absolute bandwidth, for example 3 Hz, 10 Hz etc. and the constant percentage bandwidth filter where the filter bandwidth is a constant percentage of the tuned centre frequency, for example 3%, 10% etc. Constant Bandwidth or Constant Percentage Bandwidth Frequency Analysis? | Required changes are done.  [Page no. 16 & 19; Line no. 356 to 357; 375 to 376]  Required changes are done.  [Page no. 21 & 24; Line no. 419 to 420; 439 to 440] | Thank you for your insightful observation. In our analysis, **constant bandwidth frequency analysis** was employed for the entire frequency range of **0 to 100 Hz**. This approach was chosen because it provides uniform resolution across the low-frequency range, which is particularly suitable for identifying vibration characteristics and tool wear patterns within this bandwidth. Given the relatively narrow and lower frequency spectrum of interest, constant bandwidth filtering ensures better interpretability and consistency in the analysis compared to constant percentage bandwidth, which tends to offer diminishing resolution at lower frequencies. **[Figures 8 and 11; Figures 13 and 16]** |
| 6 | An important property of modes is that any forced or free dynamic response of a structure can be reduced to a discrete set of modes. The modal parameters are: • Modal frequency • Modal damping • Mode shape. Please designate. | Required changes are done.  [Page no. 15; Line no. 331 to 332] | Thank you for your valuable observation. You are absolutely right that any forced or free dynamic response of a structure can be represented through a discrete set of modes characterized by modal frequency, damping, and mode shape. In our study, a finite element model was developed and fine-tuned using damping and other relevant parameters. The resulting modal frequencies and associated mode shapes were then compared with the experimental test results, as presented in Table 3. |
| 7 | Ideally a mobility measurement should simply involve exciting the structure with a measurable force, measuring the response, and then calculating the ratio between the force and response spectra. In practice however, we are faced with a number of problems: • Mechanical noise in the structure, including non-linear behaviour • Electrical noise in the instrumentation • Limited analysis resolution To minimize these problems we have to apply some statistical methods to decide how to estimate an FRF from our measurements. Estimation from data containing random noise generally involves some form of averaging. What techniques can we use for averaging the output/input ratio? | Required changes are done.  [Page no. 20 & 25; Line no. 403 & 468] | Thank you for your valuable comment. We acknowledge the importance of addressing noise and resolution limitations when estimating Frequency Response Functions (FRFs). In the present study, a simple statistical approach was employed to manage these practical challenges. Specifically, averaging techniques were applied to extract meaningful values from the measured data:   - To identify the **minimum stiffness values**, measurements were statistically averaged within the frequency range of **20–60 Hz** (as presented in **Table 4** and **Figure 12**). - Similarly, to determine the **maximum vibration amplitudes**, the data within the **0–100 Hz** range were statistically analyzed (refer to **Table 5** and **Figure 17**).   These statistical evaluations allowed us to mitigate random noise effects in both input and output signals. Furthermore, to derive the **optimum thickness**, a **linear interpolation method** was used based on the trend of the processed data. While more complex FRF estimation methods (such as Welch’s method or cross-spectrum averaging) can be considered in future work, the selected approach was found sufficient for the objectives of this study. |
| 8 | Please compare your research results with those of other scientists. | Required changes are done.  [Page no. 15, 20 & 25; Line no. 331 to 332, 403 & 468] | We appreciate the reviewer’s suggestion. In general, thickness optimization in existing literature has primarily been performed using CAE simulation software or through iterative sensitivity studies, which often involve time-consuming trial-and-error procedures. In contrast, our work introduces novel and efficient methods for determining optimum thickness:   - A linear trend line equation was established for each bending mode based on modal frequency analysis, which allows direct estimation of optimum thickness without iterative simulations. - Additionally, dynamic stiffness (refer Table 4 and Figure 12) and vibration response analysis (refer Table 5 and Figure 17) were used to derive an interpolation-based method for estimating the required thickness.   These approaches not only reduce computational effort but also provide reasonably accurate estimates, as compared to the detailed simulations commonly found in prior studies. This comparison highlights the practicality and effectiveness of our methods over traditional approaches. Relevant discussions have now been included in the revised manuscript to better contextualize our results with respect to previous work. |

| ***Reviewer #2*** | | | |
| --- | --- | --- | --- |
| **Sr. No.** | **Instructions and queries** | **Corrections/modifications made as per instructions and queries** | |
|  |  | **Status** | **Detailed description (All changes are shown by blue colour)** |
| 1. | (I) Document. Authors must take care with written language so they can convey what they really want. The importance of content deserves more careful and technical language. | Required changes are done. | We sincerely thank the reviewer for highlighting the importance of using precise and technical language. We have carefully revised the manuscript and made substantial improvements to the overall language, grammar, and clarity to ensure that the content is conveyed more effectively and professionally. We have done our level best to enhance the technical tone and accuracy of the text throughout the manuscript. |
| 2. | (R) Abstract. The abstract should be more succinct. | Required changes are done.  [Page no. 1 & 2; Line no. 20 to 42] | Thank you for the valuable suggestion. The abstract has been revised to be more concise while retaining the key objectives, methodology, and findings of the study. |
| 3. | (R) Keywords. Authors should avoid using words used in the title for broader content search. | Required changes are done.  [Page no. 2; Line no. 43] | Thank you for the valuable suggestion. In response, the keywords have been carefully reviewed and revised. Some of the original keywords have been modified or replaced with broader and more diverse terms that are not directly repeated from the title, to enhance content discoverability and align with indexing guidelines. |
| 4. | (I) Page 2, Introduction, first sentence. Before the COVID-19 pandemic, were cars not commonly used? (reference to 1.) | Required changes are done.  [Page no. 2; Line no. 45 to 47] | We appreciate the reviewer’s observation. We agree that cars were commonly used even before the COVID-19 pandemic. However, the intention of the statement was to emphasize the increased reliance on personal vehicles, especially cars, after the pandemic due to health and safety concerns. The following sentence is modified:   - After the COVID-19 pandemic, cars are increasingly used in both metropolitan cities and small towns. The car can provide transportation and isolation for a small family. |
| 5. | (I) Page 2, Introduction, third sentence. “The car door is used to get in and out of the car while it is in use”. What do the authors mean? Getting in and out while the car is moving? (reference to 1.) | Required changes are done.  [Page no. 2; Line no. 45 to 47] | Thank you for pointing this out. The sentence “The car door is used to get in and out of the car while it is in use” has been removed in the revised manuscript to avoid any confusion or ambiguity. |
| 6. | (R) Authors should improve the state of the art. | Required changes are done. | Thank you for your valuable suggestion. In response to your comment, the state-of-the-art section has been thoroughly revised and updated in the revised manuscript. Recent and relevant literature has been incorporated to provide a more comprehensive overview of the current advancements related to the topic. The discussion has also been enhanced to highlight the existing research gaps more clearly and to better position the current study within the context of recent developments. |
| 7. | (R) Symbols should be italicised. | Required changes are done. | Thank you for the suggestion. The required changes have been made, and all symbols have been italicised appropriately in the revised manuscript. |
| 8. | (I) In equations, all symbols must be defined. | Required changes are done.  [Page no. 6, 7, 8] | Thank you for your observation. All symbols used in the equations have now been clearly defined in the revised manuscript to enhance clarity and understanding. |
| 9. | (R) In figures with more than one image, they must have their own identifiers [(a), (b),...] and be properly identified/described in figure’s caption. | Required changes are done.  [Page no. 14; Line no. 318] | The required changes have been made in the revised manuscript. All figures containing multiple images now include appropriate sub-figure identifiers [(a), (b), ...] and have been properly described in the respective figure captions. For. Ex.; Figure 6. |
| 10. | (R) Table 2. Authors should better define the units in the second column. | Required changes are done.  [Page no. 12; Line no. 282] | Thank you for the observation. A separate column specifying the units for Modulus of Elasticity and Speed of Sound has been added in Table 2 for better clarity and definition. |
| 11. | (R) Figures 8, 9, 10, 11, 13 and 16. Y-axis values must be the same on graphs, for better comparison. | Required changes are done.  [Page no. 16, 17, 18, 19, 21 & 24] | Thank you for the suggestion. The Y-axis values for Figures 8, 9, 10, 11, 13, and 16 have been standardized to ensure consistency and facilitate better comparison. The revised manuscript reflects these changes. |
| 12. | (R) All figures must be named in the text before they appear. | Required changes are done. | Thank you for the observation. As suggested, all figures are now properly cited in the text before they appear in the revised manuscript. Necessary alignments have been made for all applicable figures, excluding those under result presentation where sequential context is maintained. |
