# Supplementary material for: Optimization of inner panel thickness for enhanced stiffness and vibration control in car door assemblies
Source: PLoS One. 2025 Nov 13;20(11):e0331318. doi: 10.1371/journal.pone.0331318 (PMC12614550; doi:10.1371/journal.pone.0331318)
Supplement: Attachment — Submitted filename: PONE-D-25-18089_Revision 2_Responses.docx [file pone.0331318.s004.docx]

**Responses to the Review Comments**

| ***Reviewer #2*** | | | |
| --- | --- | --- | --- |
| **Sr. No.** | **Instructions and queries** | **Corrections/modifications made as per instructions and queries** | |
|  |  | **Status / Page** | **Detailed description** |
| 1 | (R) Document. Authors must take care with written language. The importance of content deserves more careful and technical language. There was a noticeable improvement in writing. The document is understandable despite being written in informal language. | Page 3, 4, 8, 9 | Thank you for your valuable feedback. We have carefully revised the manuscript to enhance the technical language and overall writing quality, ensuring the content is clear, precise, and appropriately formal. |
| 2 | (R) Document. Change “...between... to…” to “...between... and...”. | Page 3 | The correction from “between... to...” to “between... and...” is grammatically appropriate, as “between” should pair with “and” when referring to two elements. The change has been accepted and incorporated. |
| 3 | (S) Keywords. Authors should avoid using words used in the title for broader content search. For example (it is not mandatory to use these examples): “dynamic analysis”, “FEM”, “modal frequency”. | Page 2 | As suggested, the keywords have been revised to avoid repetition of terms already used in the title and to enhance the manuscript's discoverability in broader content searches. The updated keywords — Car Door NVH, FEA, FRA, Dynamic Analysis, Modal Frequency Analysis — now provide a balanced blend of specificity and breadth while aligning with the core technical themes of the paper. |
| 4 | (S) Line 68. There is a disagreement in the requirement to separate the symbol "%" from the respective value. In my opinion, it should be separated. | Page 3 | As per standard scientific and technical writing conventions (e.g., SI units guidelines and style manuals like ISO/IEC and APA), a non-breaking space is recommended between a number and the percentage symbol. Hence, the correction has been made as “5.74 %” to align with these formatting standards. |
| 5 | (R) Lines 70, 76, 99, 102, 112, 117, 118, 120, 123, 125, 167. The sentence needs to be improved. Authors should begin the sentence with "AUTHOR studied/analysed/..." | Page 3, 4, & 5 | We thank the reviewer for highlighting the need to improve sentence structure for clarity and consistency. As suggested, we have revised the mentioned lines to begin with action-oriented phrases such as “The authors studied…”, “The authors analysed…”, etc. This improves the readability, maintains an active voice, and clearly reflects the authors’ contribution throughout the manuscript. The necessary modifications have been incorporated accordingly. |
| 6 | (R) Line 108. Change “it’s” to “its”. | Page 4 | Required changes are done. |
| 7 | (R) Line 163. Remove the worth “the”. | Page 6 | Required changes are done. |
| 8 | (R) Figures 2, 6, 8-11, 13-16. In figures with more than one image, they must have their own identifiers [(a), (b),] and be properly identified/described in figure’s caption and text. | Figure 2  Page 7,  Page 15 – 18,  Page 20 - 23 | - Figure 2 has been updated as per the comment. - Figure 6 already includes the identifiers and corresponding descriptions in the caption: (a) Schematic and (b) Experimental Test Set-up. - Figures 8–11 and 13–16: Each sub-image is titled as Dx (e.g., D8, D9, etc.), and both the figure titles and captions are consistently referenced and explained in the manuscript text, maintaining clarity and proper identification. |
| 9 | (I) Line 187. The sentence needs to be improved. | Page 7 | Required changes are done. |
| 10 | (R) Line 191. Change “1.00” to “1.0”. | Page 7 | Required changes are done. |
| 11 | (R) Line 192. Change “steps of 0.2 mm (1.0, 1.2, 1.4, 1.6, 1.8, 2.0, 2.2, and 2.4 mm)” to “steps of 0.2 mm, (1.0, 1.2, 1.4, 1.6, 1.8, 2.0, 2.2 and 2.4) mm”. | Page 7 | Required changes are done. |
| 12 | (I) Line 237. “… were taken in all three XY directions…”. Three (XYZ) or two (XY) directions? Which direction Y is? | Page 9 | Thank you for the observation. The sentence has been corrected to "in all three XYZ directions" to accurately reflect the three-dimensional measurement. Direction Y refers to the vertical axis in the experimental setup. The change has been implemented in the revised manuscript.. |
| 13 | (R) Table 2. SI density units: [kg/m^3] | Page 11 | Thank you for your observation. You are right that [kg/m³] is the SI unit for density. However, the unit used in the Hypermesh software environment is tonnes/mm³, which is consistent with the solver’s default unit system. This unit was maintained throughout the model setup to ensure compatibility with software input requirements. For clarity, this has now been mentioned in the revised Table 2. |
| 14 | (R) Table 3. Lateral, vertical and torsional bending units? | Page 14 | The title of Table 3 has been updated to include the unit of frequency as Hz to clearly indicate that the lateral, vertical, and torsional bending modes are expressed in terms of natural frequencies. This addresses the reviewer’s concern regarding missing units. |
| 15 | (R) Figures 8 to 12. SI stiffness units: N/m. You can solve by changing the axis title to “Dynamic stiffness (x 10^6 N/m)”. | Page 15 - 20 | Thank you for the observation. You are right that **N/m** is the standard SI unit. However, as per common industrial practice, **MN/m** is typically used for representing dynamic stiffness values without additional multipliers, which is why Figures 8 to 11 follow this format. In Figure 12, the stiffness values are comparatively lower, and using **N/m** improves clarity and avoids the need for decimal values. This was done purely for better visual presentation and consistency in data interpretation. |
| 16 | (R) All figures must be named in the text before they appear. | Page 20 - 23 | We have ensured that all figures are appropriately aligned and referred to in the text before their appearance, particularly for result-related figures. The interpretations and discussions in the manuscript are structured to follow the logical flow of the results as presented through the figures. Non-result figures (e.g., schematic diagrams or setup illustrations) are also placed close to their initial reference in the text for clarity and coherence. |
| 17 | (R) Lines 351 and 352. change “(T10, to T24)” to “(T10 to T24)” and “(D1, to D15)” to “(D1 to D15)”. | Page 15 | Required changes are done. |
| 18 | (S) Line 385. Improving English writing. For example, “The following are the interpretations after an…”. | Page 18 | Required changes are done. |
| 19 | (R) Line 489. Improving English writing. “… in Tables 3, 4, and 5.” or “… in Tables 3 to 5.” | Page 25 | The phrase “in Tables 3, 4, and 5” is preferred for clarity and precision, especially in academic writing. It explicitly lists each table, avoiding ambiguity. While “in Tables 3 to 5” is grammatically acceptable and often used for consecutive references, the former format aligns better with formal and unambiguous scientific writing standards. |
| 20 | (I) In finite element analysis, what is the dimension of the finite elements? For a good analysis, there should be at least 7 finite elements at one wavelength (there is bibliography on this subject). Has this been verified or has any sensitivity analysis been done on the finite element dimension? | Page 10 & 29 | - In our finite element model, the unit system adopted is [N mm s tonne], which aligns with practical industrial standards, although the base unit system of Hypermesh is [N m s kg]. - For accurate representation of wave propagation, it is commonly accepted that at least six elements per wavelength are necessary. Accordingly, we have ensured adequate mesh refinement in our model. - The element size and type selection have been guided by established industrial practices and validated through the referenced technical paper [31]. - Furthermore, to address the sensitivity related to element dimensions, a correlation study of the door assembly finite element model has been performed, as summarized in Table 3, confirming the reliability and accuracy of the chosen mesh density. |
