# Supplementary material for: Optimization of inner panel thickness for enhanced stiffness and vibration control in car door assemblies
Source: PLoS One. 2025 Nov 13;20(11):e0331318. doi: 10.1371/journal.pone.0331318 (PMC12614550; doi:10.1371/journal.pone.0331318)
Supplement: Attachment — Submitted filename: Responses_3_to_Reviewers_Sheet_27July2025.docx [file pone.0331318.s005.docx]

**Responses to the Review Comments**

| ***Reviewer #2*** | | | |
| --- | --- | --- | --- |
| **Sr. No.** | **Instructions and queries** | **Corrections/modifications made as per instructions and queries** | |
|  |  | **Status / Page** | **Detailed description** |
| 1 | Page 3, paragraph 4; | Page 3 | - XIQUAN QI et al [4] verified the finite element modal analysis results through an experimental test conducted within a semi-anechoic chamber. |
| 2 | page 4, last sentence of paragraph 1, | Page 4 | - A. Lucifredi et al [12] demonstrated a good correlation between the door noise pressure and vibro-acoustic analysis and an experimental test. |
| 3 | 1st sentence of paragraph 2 | Page 4 | - D. A. Desai et al [13] analyzed interior noise of a simplified model of a car tested in an anechoic chamber and is compared with vibro-acoustic FEA. |
| 4 | 3th sentence of paragraph 3 | Page 4 | - Mihir H. Patel et al [20] carried out an analysis of variable sample data of real-time test using FFT to find fundamental frequencies. |
| 5 | Page 5, last sentence of paragraph 1 | Page 5 | - P. M. Jadhav et al [28] established the correlation of test and CAE results and isolator design for door vibration improvement. |
| 6 | Additional revision –  page 4, last sentence of paragraph 4 | Page 4 | - Rui Feng et al [25] used a reanalysis method, and an algorithm was developed using initial parameters and used to analyze and optimize the new structure of the door. |
|  |  |  |  |
|  |  |  |  |
|  |  |  |  |
